# Effects of Hydroalcoholic Extract from Salvia *verticillata* on Pharmacological Models of Seizure, Anxiety and Depression in Mice

**Published:** 2011

**Authors:** Nima Naderi, Nida Akhavan, Farzad Aziz Ahari, Nina Zamani, Mohammad Kamalinejad, Mohammad Shokrzadeh, Nematollah Ahangar, Fereshteh Motamedi

**Affiliations:** a *Neuroscience Research Center, Shahid Beheshti University of Medical Sciences, Tehran, Iran.*; b *Faculty of Pharmacy, Mazandaran University of Medical Sciences, Sari, Iran. *; c *Neuroscience Research Center, Shahid Beheshti University of Medical Sciences, Tehran, Iran.*; d *Faculty of Pharmacy, Shahid Beheshti University of Medical Sciences, Tehran, Iran.*

**Keywords:** *Salvia verticillata*, Anxiety, Depression, Seizure, Mice

## Abstract

*Salvia verticillata *is one of the salvia species which possesses remarkable antioxidant activity. In the present study, we investigated the possible effects of hydro-alcoholic extract from *Salvia verticillata *plant (SVE) in various models of anxiety, depression and seizure in mice. Mice were randomly divided into control (saline), SVE-treated and standard treatment groups. The SVE-treated groups received oral administration of various doses of SVE. As a standard treatment, diazepam and imipramine were used orally for anxiety/seizure and depression tests, respectively. The results of the study revealed that the plant extract produced significant anticonvulsant activity in maximal electroshock and pentylenetetrazol induced seizure models. Moreover, in forced swim test and tail suspension test of depression, SVE produced significant antidepressant effect in mice compared to control group. However, SVE did not show any effects on anxiety-like behavior of mice in elevated plus maze and light-dark tests. These results suggest potential therapeutic effects of the plant extract in seizure and depression.

## Introduction

There are many drugs available for the treatment of patients with various neurological and psychological disorders; however, overall clinical outcomes and the standard of care for most patients are still far from optimal. Current treatments have modest efficacy and many patients do not respond or are unable to tolerate pharmacological approaches. Therefore, the medical need for newer, better-tolerated and more efficacious treatments remains high. Herbal medicines which include a range of pharmacologically active compounds can provide an alternative treatment or be used to enhance the effect of current medications used for treatment of different mood and neurological disorders.

Salvia is the largest genus of plants in the mint family Lamiaceae. The genus is distributed throughout the world, with the center of diversity and origin appearing to be Central and South Western Asia (1). Salvia species are used to treat various conditions including respiratory diseases (colds, coughs and bronchial infections), gastrointestinal diseases (colic, diarrhea, indigestion and abdominal trouble), hepatitis and other hepatic sicknesses, cardiovascular diseases, infections, cancer, inflammation, loss of memory, menstrual disorders, miscarriage and insomnia (2). The curative properties of Salvia species and their isolated constituents are mainly due to the possession of significant antioxidant activities in enzyme-dependent and enzyme-independent systems (3). 

*Salvia verticillata *L. (purple rain or lilac sage) was first described by Carolus Linnaeus in 1753. It is used locally in folk medicine and as a garden ornamental. It has been reported as an antioxidant and acetylcholinesterase inhibitor (4, 5). The plant contains a variety of polyphenols, volatile oils and diterpenoids (6, 7). The essential oils of *Salvia verticillata *have shown moderate to high inhibitory activity against bacteria (8) and *Salvia verticillata *may have the potentiality of being used in lost or declining cognitive functions (9). In a spectrophotometric method based on the reduction of the stable DPPH free radical, it was suggested that *Salvia verticillata *can be considered as a natural source of radical scavenger (4, 10). Radical scavenging activity is the ability to protect cells from different kinds of oxidative stress. Excessive production of free radicals has been implicated in the pathogenesis of considerable ranges of neurological disorders including epilepsy and oxidative stress may be a common pathogenic mechanism underlying many major psychiatric disorders such as anxiety and depression (11). The aim of the present study was to evaluate the effects of *Salvia verticillata *on epilepsy, anxiety and depression.

## Experimental

*Animals*

This study was performed on male NMRI mice weighing 20-30 g (Pasteur Institute, Tehran, Iran). The animals were housed in colony cages (7-8 mice per cage) with free access to food and tap water and under standardized housing conditions with a 12 : 12 h light-dark cycle (lights on at 7:00 AM) and temperature-controlled environment (22 ± 1°C). 

*Plant collection and preparation of hydroalcoholic extract*

The plant material was collected from Gachsar region (Tehran, Iran). The plant was identified and authenticated by M. Kamalinejad of the Department of Pharmacognosy, Faculty of Pharmacy, Shahid Beheshti University of Medical Sciences (Tehran, Iran). The voucher specimen coded S-760 has been deposited at the herbarium of the Faculty of Pharmacy, Shahid Beheshti University. The plant extract was obtained using maceration method: 100 g of dried plant was soaked in 1 L of water : ethanol (1 : 1) for 48 h. The resulted solution was then filtered using a filter paper and transferred to a rotary evaporator apparatus for further extraction.

*Chemicals*

Pentylenetetrazole (PTZ), diazepam and imipramine were purchased from Sigma Chemical Company (St Louis, MO, USA). PTZ and imipramine were dissolved in saline. Diazepam was suspended in normal saline. *Salvia verticillata *hydroalcoholic extract (SVE) was diluted in distilled water. All the solutions were freshly made on the day of testing and administered to a final volume of 10 mL/Kg body weight of mice.

*Study design*

All experiments were performed according to the Principles of Laboratory Animal Care (NIH publication #85-23, revised in 1985). Animals were removed from the colony room to the testing room in their home cages and allowed to adapt to the new environment for at least 2 h before testing. Each mouse was used only once. All tests were conducted between 10:00 AM to 4:00 PM. Animals were randomly assigned to different experimental groups consisting of ten mice. SVE, diazepam and imipramine were administered per-oral*. *After receiving the appropriate dose of compounds, animals were returned to a second cage where they remained for 30 min before the evaluating respective behavioral test.

*Open field motor activity test*

In view of the fact that changes in motor activity could result in false findings in the elevated plus-maze test, light-dark test, forced-swim test and tail suspension test, investigation of the effects of SVE and standard positive compounds on locomotion of mice were undertaken. Locomotor activity of mice was assessed in Plexiglas locomotor activity chambers (40 × 40 × 20 cm) in a lighted room. The mice were placed individually at the center of the arena 30 min after the oral administration of the test compounds and the locomotion was recorded during a 5-min session using a video camera that was positioned above the apparatus and connected to a computer. Then, data were analyzed using EthoVision^®^ software (version 3.1, Noldus Information Technology, The Netherlands). The distance moved (cm) during the recording session was measured. A decrease in total distance moved by animals in treatment groups compared to control group was interpreted as a decrease in motor activity.

*Elevated plus maze test*

Activity and anxiety-related behaviors were assessed using the mouse elevated plus maze (EPM) test. The procedure was similar to that described by Dawson and Tricklebank (12), with minor modifications. The apparatus consists of two open and two enclosed horizontal perpendicular arms (30 × 5 cm) positioned 40 cm above the floor. The junction of four arms forms a central square platform (5 × 5 cm). The experiments were carried out in a sound attenuated room. The environment was illuminated by two 40 W white fluorescent lights placed 3 m away from the EPM. Drugs were given 30 min before submitting the animal to the EPM apparatus. Each animal was placed in the central platform facing one of the open arms and allowed to explore freely for 5 min. The maze was thoroughly cleaned between each trial with 10% ethanol solution and afterwards, by a dry cloth. The sessions were recorded by a camera and data were obtained using EthoVision^®^ software as described above. During the 5 min trial, the behavior of each mouse was recorded as: (I) the number of entries into the open or closed arms; (II) average time spent by mouse in each of the arms. The number of entries into open arms (OAE) and the time spent in open arms (OAT) were expressed as percentages of total entries and total test time, respectively (*i.e. *OAE% and OAT%). The increase in both OAT% and OAE% has shown to be an index of lowered anxiety behavior. The number of entries into closed arms (CAE) is an index of animal activity.

*Light-dark test*

This test also evaluates the anxiolytic activity of compounds. For the light-dark test, we followed the procedure described by Crawley and Goodwin (13). The apparatus consisted of a dark plastic compartment covering 1/3 (16 × 50 cm) and a light compartment covering 2/3 (34 × 50 cm) of the testing area. The light compartment was illuminated under an 80 W light. The dark one received only a part of the room illumination. The two parts were connected by a central opening (7 × 7 cm) on floor level. The animals were allowed to explore the light-dark arena 30 min after receiving SVE or diazepam (as a standard anxiolytic compound). The behavior of each mouse was then tracked for 5 min and the number of entrance into the light compartment as well as the time spent in the light compartment of the apparatus were measured. An increase in mentioned parameters was considered as the anxiolytic effect of compound.

*Tail suspension test*

This assay detects the activity of antidepressants and is used here as a modification of the procedure of Steru’s method (14). Mice were securely fastened with medical adhesive tape by the tip (1-2 cm) of the tail to a metal hook and suspended by their tails in a visually isolated cubicle. Immobility, defined as the mice hanging passively without movements to right themselves, were recorded during a 6 min trial. SVE in different doses was given 30 min before the test. Imipramine (15 mg/Kg, p.o., 30 min prior) was used as a comparator standard. 

*Forced swim test*

This experiment was also carried out to evaluate the antidepressant effects of test and standard compounds. The forced swim test was performed using the original method described by Porsolt *et al*. (15). The forced swim chamber was a 2000-mL Pyrex beaker with a diameter of 14 cm filled with tap water (25°C) to a depth of 14 cm. To optimize the contrast, a black background was used behind the beaker. Mice were transferred to the beaker 30 min after drug administration and the animal behavior was observed for a period of 6 min. The duration of immobility was recorded during the last 5 min of a 6 min trial. A mouse was regarded as immobile when floating motionless or making only those movements necessary to keep its head above the water. The water in the beaker was changed between each trial.

*Pentylenetetrazole (PTZ) induced seizure test*

In the PTZ-induced seizure test, mice treated with either SVE or standard anticonvulsive substance (diazepam) were challenged with a subcutaneously (s.c.) administered convulsive dose (CD_80_) of PTZ (85 mg/Kg) and then placed in isolation cages and observed for the next 60 min for the presence or absence of an episode of clonic spasms persisting for at least 10 sec. Animals not displaying a minimally clonic seizure were considered protected. In order to evaluate the anticonvulsant effect of *Salvia verticillata*, five doses of SVE were administered orally to groups of 10 mice, 30 min before the PTZ injection. Diazepam (0.1, 1, 2 and 4 mg/Kg; p.o.) was used as positive control and administered 30 min before the PTZ injection.

*Maximal electroshock seizure (MES) test*

Electroshock was produced by means of an alternating current (fixed current intensity = 40 mA, pulse duration = 0.2 sec, frequency = 50 Hz, pulse width = 0.5 msec) through ear clip electrodes by a generator (ECT unit 57800, Ugo Basile, Italy). The criterion for the occurrence of seizure activity was the hind limb tonic extension (HLTE) (16, 17). This stimulus was sufficient to produce hind limb tonic extension in control animals. The animals (in groups of 10) were administered five doses of SVE to calculate the anticonvulsant effect. The MES test was performed 30 min after the SVE administration. Animals not displaying electroshock induced HLTE were considered protected. Diazepam (0.25 and 2.5 mg/Kg) was used as a positive control and administered p.o. 30 min before the electroshock test.

*Statistical analysis*

Data are represented as group means + standard error of mean (SEM). Data were analyzed using one-way analysis of variance (ANOVA) followed by Dunnett’s post test and levels at p < 0.05 were considered significant. For calculation the ED_50_ dose of compounds to protect mice against MES or PTZ seizure, probit analysis has been used and ED_50_ with 95% confidence intervals were reported. Statistical analysis was performed using SPSS (ver. 13, SPSS Inc.).

## Results and Discussion

The amount of dried extract that was used in this study contained 15% (W/W) of plant material.

*Locomotor activity*

**Figure 1:**
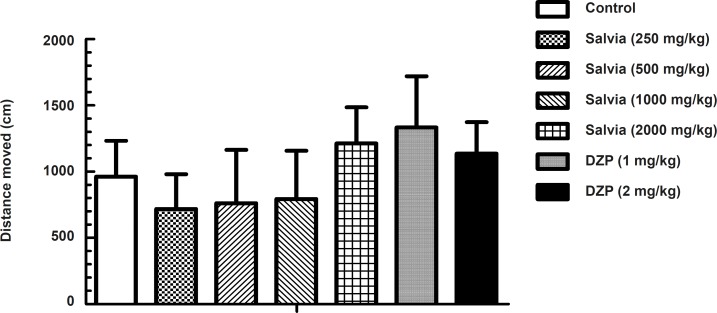
Spontaneous locomotor activity of mice treated with various doses of SVE and diazepam. Each group contains 10 mice. The test was performed 30 min after treatment. Total distance moved (cm) in 5 min trial was measured by Ethovision^® ^software. Data are expressed as mean + SEM

The explorative drive and motor behavior in a novel environment was investigated in the open field paradigm (Figure 1).

 Total distance moved was taken as the parameter of activity in an unfamiliar environment. Although statistical analysis by one-way ANOVA indicated significant difference in total distance moved between experimental groups (*F*_6, 63 _= 5.655, p < 0.001), post-hoc analysis by Dunnett’s test revealed no significant change in total distance moved between mice treated with either SVE or diazepam compared to control (saline) group.

*Elevated plus maze*

**Figure 2:**
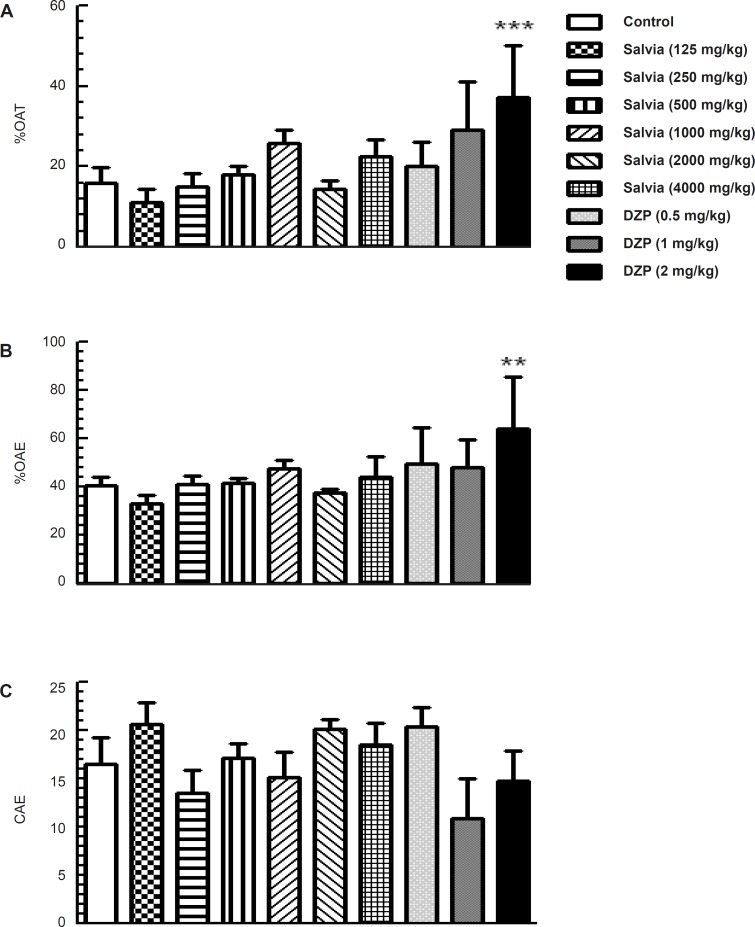
Effect of oral administration of SVE or diazepam on anxiety-like behaviour of mice in the elevated plus maze test. SVE or diazepam was administered 30 min before the test. Control group received saline 30 min before the test. The animal behaviour was evaluated for a period of 5 min. Columns represent the means + SEM of (A) percentage of time spent in open arms, (B) percentage of open arm entries and (C) number of close arm entries in 10 mice. ** p < 0.01, *** p < 0.001 significantly different from control group (Dunnett’s test).

Analysis of the elevated plus maze data using one-way ANOVA revealed significant difference among experimental groups in the main parameters indicative of anxiety-like behaviors such as percentage of time spent in the open arms (*F*_9,70 _= 5.316; p < 0.001) and the percentage of open arm entries (*F*_9,70 _= 4.239; p < 0.001). However, further analysis by Dunnett’s test revealed no significant change in groups of mice treated with various doses of SVE (125, 250, 500, 1000, 2000 and 4000 mg/Kg) compared to control group. A significant difference was observed in percentage of time spent in the open arms (p < 0.001) and the number of open arm entries (p < 0.01) in mice treated with diazepam (2 mg/Kg) compared to control group (Figure 2A and 2B). Parameter reflecting changes in locomotor activity in this model (the number of closed arm entries) did not differ between groups (*F*_9, 70 _= 1.628, *p = *0.124; Figure 2C). 

*Light-dark test*

**Figure 3:**
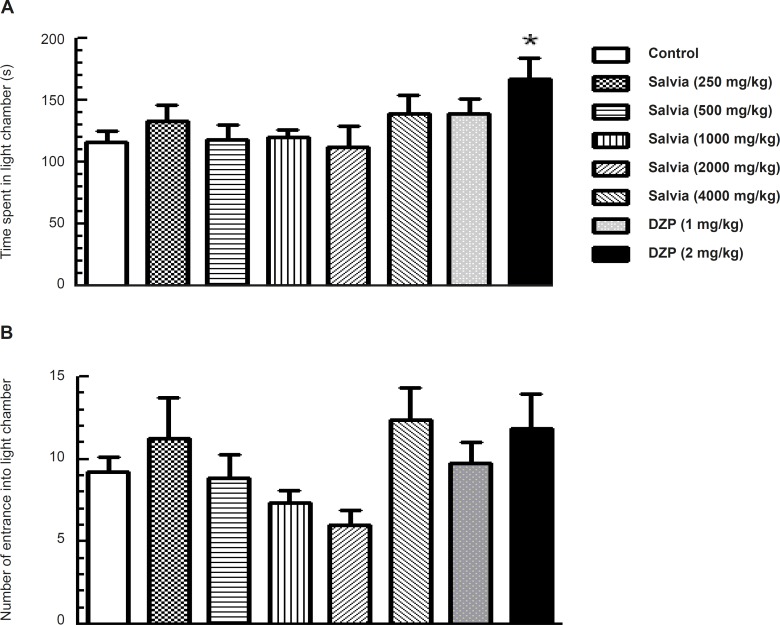
Effect of oral administration of SVE or diazepam on the behaviour of mice in Light–dark test. SVE or diazepam was administered 30 min before the test. Control group received saline 30 min before the test. The animal behaviour was evaluated for a period of 5 min. The upper (A) and lower (B) panels depict the effects of SVE or diazepam (DZP) on time spent and number of entrance into light chamber, respectively. Each bar represents the mean value + SEM of 10 mice. * P < 0.05 significantly different from control group (Dunnett’s test).

One-way ANOVA indicated significant difference among the experimental group for time spent in light compartment (*F*_7, 53_ = 2.297, *p *= 0.04; Figure 3A), but no significant difference for the number of entrance into light chamber (*F *_7, 53_ = 1.983, *p *= 0.074; Figure 3B). Dunnett’s post-hoc test revealed that the groups of mice that received various doses of SVE showed no significant change in time spent in the light compartment compared to control group. However, for diazepam (2 mg/Kg) treated mice, a significant increase was observed in time spent in the light compartment compared with control group (p < 0.05; Figure 3A).

*Forced swim test*

**Figure 4:**
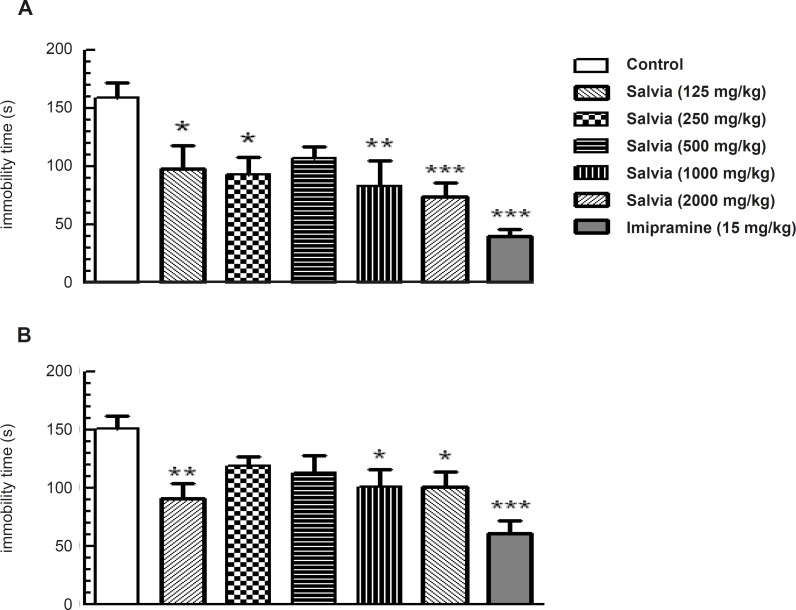
SVE reduced immobility time in the mouse forced swim test (A) and tail suspension test (B). This reduction was similar to that produced by acute administration of imipramine. Mice were pretreated orally with either imipramine or various doses of SVE, 30 min prior to being subjected to test. Bars represent mean + SEM of 10 animals per group. * p < 0.05, ** p < 0.01, *** p < 0.001 significantly different from control group (Dunnette’s test).

To understand the potential role of SVE in models used to predict antidepressant effects, we evaluated the effects of plant extract in the mouse forced-swim test. One-way ANOVA revealed significant change in immobility time among the experimental groups (*F*_6,62 _= 5.532, *p = *0.001) that was statistically significant at doses of 125 mg/Kg (p < 0.05), 250 mg/Kg (p < 0.05), 1000 mg/Kg (p < 0.01) and 2000 mg/Kg (p < 0.001) compared to control group. The group received the standard antidepressive compound imipramine (15 mg/Kg) also showed significant reduction in immobility time (p < 0.001) compared to control group (Figure 4A).

*Tail suspension test*

Further substantiating the antidepressant-like effects of plant extract, we also evaluated the effects of SVE in tail suspension test. One-way ANOVA revealed a significant difference in immobility time among the experimental groups (*F*_6, 63 _= 4.745, p < 0.001; Figure 4B). Post-hoc analysis revealed that oral administration of SVE, 30 min before the test, produced a significant reduction in the immobility time at the doses of 125 mg/Kg (p < 0.01), 1000 mg/Kg (p < 0.05) and 2000 mg/Kg (p < 0.05) compared to control group. Furthermore, imipramine (15 mg/Kg) significantly decreased immobility time (p < 0.001) compared to control group (Figure 4B).

*PTZ-induced seizure test*

The results are presented in Table 1. 

**Table 1:** Anticonvulsant effects of oral administration of SVE or diazepam on PTZ-induced seizures. The test was performed 30 min after drug administration to mice. Mice in control group were treated with saline. Data represent the percentage of mice which did not show tonic-clonic seizure during 60 min after PTZ administration (n = 10)

**Dose (mg/Kg)**	**Protection (%)**
**Control**	20
**SVE**	
125	20
250	40
500	50
1000	80
2000	100
**Diazepam**	
0.1	20
1	60
2 4	80 100

Pretreatment with SVE protected mice against PTZ-induced seizures dose-dependently. SVE administration at 125, 250, 500, 1000 and 2000 mg/Kg produced 20%, 40%, 50%, 80% and 100% protection, respectively. In order to measure the relationship between the dose of SVE and the proportion of mice not exhibiting a seizure response to PTZ injection, probit analysis was performed and the ED_50 _of 360 mg/Kg (with 95% confidence intervals between 205 and 575 mg/Kg) was calculated for protective effect of SVE against PTZ-induced seizure. The standard anticonvulsant drug diazepam at the doses of 0.1, 1, 2 and 4 mg/Kg produced 20%, 60%, 80% and 100% protection against PTZ-induced seizure. Probit analysis revealed an ED_50_ of 0.454 mg/ Kg (with 95% confidence intervals between 0.139 and 0.926 mg/Kg) for protective effect of diazepam against PTZ-induced seizure. 

*Maximal electroshock seizure test*

SVE produced dose-related anticonvulsant activity against electroshock-induced hind limb tonic extension phase. As shown in Table 2, SVE at the dose of 250, 500, 750, 1000 and 2000 mg/Kg produced 30%, 40%, 70%, 70% and 80% protection. Higher doses did not show further protection against electroshock-induced seizure. 

**Table 2:** Anticonvulsant effects of oral administration of SVE or diazepam on electroshock-induced seizure. The test was performed 30 min after drug administration to mice. Mice in control group were treated with saline. Data represent the percentage of mice which did not show hind-limb tonic extension (HLTE) after MES (n = 10

**Dose (mg/Kg)**	**Protection%**
**Control**	0
**SVE**	
250	30
500	40
750	70
1000	70
2000	80
**Diazepam**	
0.25	30
2.5	100

Probit analysis revealed the ED_50_ of 524 mg/ Kg (with 95% confidence intervals between 144 and 910 mg/Kg) for protective effect of SVE against MES-induced seizure. The standard anticonvulsant drug diazepam, at the doses of 0.25 and 2.5 mg/Kg produced 30% and 100% protection, respectively. 

Despite the usage of Salvia species for treating neurological disorders in folk medicine, there is an absence of scientific reports about the effects of *Salvia verticillata *on these conditions. The results of the present study showed that pretreatment of mice with various doses of SVE produced antidepressant effects when the animals were exposed to two different depression models. A similar behavior was seen in mice treated with the standard tricyclic antidepressant agent imipramine. These models are the most generally used preclinical tests for antidepressant screening (18, 19). One concern in using the forced swim test is that the non-specific treatment effects on activity levels could complicate data interpretation. Therefore, the locomotion test was conducted in parallel with the forced swim test to identify potentially confounding effects. SVE did not alter the locomotion activity at any of the doses tested. Although much research on depression has focused on brain norepinephrine and serotonin (5-HT) systems, there is substantial evidence that other mechanisms may play important roles in the neurobiology of mood and affective disorders (20). The essential oil compositions of Lamiaceae play an important role in the ecology of these species. Like many other species of this genus, *Salvia verticillata *is a rich source of diterpenoids as well as polyphenols and volatile oils (7). *Salvia verticillata *has also a high rosmarinic acid level and there is a strong relationship between the rosmarinic acid level and antioxidant activity potential (5). Antioxidants may protect the nervous system from free radical-induced oxidative damage and they have been extensively associated with protective actions against normal and pathological cognitive declines, such as depression (21, 22). It has been demonstrated that the swimming test is sensitive to serotoninergic compounds, such as the selective serotonin reuptake inhibitor fluoxetine (23). Although other kinds of studies are obviously necessary to elucidate the mechanism of *Salvia verticillata *action in the CNS, the pattern of effects observed in the forced swim test may suggest the involvement of serotonergic brain systems on its antidepressant-like effects. There is one case study that reports the antidepressive effect from *Salvia divinorum*, another salvia species in human (24), but *in-vitro *study of Salvinorin A, one of diterpenoids derived from *Salvia divinorum*, has shown depressive-like effects (25). There is also support for the potential antidepressant activity of *Salvia elegans *(26, 27). 

Using the Elevated plus maze test, the SVE did not alter percentage of time spent and percentage of arm entries in the open arms as well as the percentage of arm entries in the closed arms. This animal model is assumed as one of the most widely validated tests for investigating the anxiolytic effects of substances (28). The anxiolytic effect was also investigated through the light-dark test, which is also useful for predicting the potency of clinically used compounds for treating anxiety. In this study, none of the groups treated with SVE showed significant change in time spent or in number of entrance into the light compartment compared to control group. There is a lack of evidence about the possible effects of *Salvia verticillata *on anxiety. There are some studies about anxiolytic effects of other salvia species. *Salvia reuterana *Boiss is demonstrated to have anxiolytic properties in the elevated plus-maze model (29). The results of a study on 30 healthy participants showed improvement in mood and cognitive performance following the administration of single doses of *Salvia officinalis *(30). Some doses of ethanolic extract of *Salvia leriifolia *showed anti-anxiety activity using EPM model (31). *Salvia elegans *has been widely used in Mexican traditional medicine for the treatment of different central nervous system diseases, principally, anxiety and scientific information provided support for the potential anxiolytic activity of hydroalcoholic extract of *Salvia elegans *(26, 27).

PTZ-induced and maximal electroshock (MES)-induced seizure models were applied to investigate the anti-epileptic properties of SVE. Systemic administration of SVE protected mice dose-dependently against seizure induced by both of these agents. The MES test is considered to be a predictor of therapeutic efficacy against generalized tonic-clonic seizures. By contrast, the PTZ test represents a valid model for human generalized myoclonic and also absence seizures (17). Therefore, it seems that the extract could be effective in these two types of human seizures. Limited information exists about anti-seizure effects of salvia species. Maklad *et al. *reported antiepileptic features from extract of *Salvia transsylvanica *(32) and extracts of *Salvia sclarea *found to possess anticonvulsive activity induced by electrical convulsions (33). As mentioned earlier, *Salvia verticillata *is a rich source of terpenoids which are reported to possess anticonvulsant activity in some experimental seizure models like PTZ, MES and electrical kindling (34). Monoterpenes also have protective effects against PTZ-, picrotoxin- and NMDA-induced convulsions (35, 36); therefore, anti-seizure activity of *Salvia verticillata *may be related to its terpenoid constituents. *Salvia verticillata *is also a source of flavonoids (37) which are known to bind to the GABA_A_-benzodiazepine site and may enhance the receptor sensitivity for endogenous GABA, which is a desired effect in the treatment of epilepsy (38, 39). As mentioned earlier, an unusually large number of useful secondary metabolites, belonging to various chemical groups such as essential oils, terpenoid compounds and phenolic derivatives, have been isolated from the *Salvia verticillata *and many of these isolated constituents possess significant antioxidant activities (5). It is concluded that antioxidants supply is important for brain functions and prevention of neurological diseases and might have neuroprotective biological targeted benefits while being used in epilepsy (11, 40).

Since changes in motor activity can have confounding characteristics on almost all tests used in this study, we evaluated the effects of SVE on locomotion applying open field test. The results of open field test showed no significant difference in locomotion activity between mice treated with SVE and the control group.

In conclusion, our results showed that SVE exerts antiepileptic and antidepressant effects in mice. However, the extract did not reveal anxiolytic properties and did not alter the motor activity. With the experimental tests used in this work which gives us information about motor activity, anxiety, depression and seizure, it is not possible to elucidate the action mechanism through which *Salvia verticillata *exerts its effects. Further studies are necessary to confirm and extend these results.
